# The genome sequence of the variegated flesh fly, *Sarcophaga variegata *(Scopoli, 1763)

**DOI:** 10.12688/wellcomeopenres.19483.1

**Published:** 2023-06-02

**Authors:** Steven Falk, John F Mulley, University of Oxford and Wytham Woods Genome Acquisition Lab, Darwin Tree of Life Barcoding collective, Wellcome Sanger Institute Tree of Life programme, Wellcome Sanger Institute Scientific Operations: DNA Pipelines collective, Tree of Life Core Informatics collective, Darwin Tree of Life Consortium

**Affiliations:** 1 Independent researcher, Kenilworth, England, UK; 2 Bangor University, Bangor, Wales, UK

**Keywords:** Sarcophaga variegata, the variegated flesh fly, genome sequence, chromosomal, Diptera

## Abstract

We present a genome assembly from an individual male *Sarcophaga variegata* (the variegated flesh fly; Arthropoda; Insecta; Diptera; Sarcophagidae). The genome sequence is 718.5 megabases in span. Most of the assembly is scaffolded into 7 chromosomal pseudomolecules including the X and Y sex chromosomes. The mitochondrial genome has also been assembled and is 18.7 kilobases in length. Gene annotation of this assembly on Ensembl identified 16,660 protein coding genes.

## Species taxonomy

Eukaryota; Opisthokonta; Metazoa; Eumetazoa; Bilateria; Protostomia; Ecdysozoa; Panarthropoda; Arthropoda; Mandibulata; Pancrustacea; Hexapoda; Insecta; Dicondylia; Pterygota; Neoptera; Endopterygota; Diptera; Brachycera; Muscomorpha; Eremoneura; Cyclorrhapha; Schizophora; Calyptratae; Oestroidea; Sarcophagidae; Sarcophaginae; *Sarcophaga*; *Sarcophaga variegata* ( Scopoli, 1763) (NCBI:txid236851).

## Background

The genus *Sarcophaga* comprises around 890 species within 169 subgenera ( Buenaventura & Pape, 2017), 36 of which have been recorded in Britain ( Whitmore *et al.*, 2020). The variegated flesh fly *Sarcophaga* ( *Sarcophaga*) *variegata* (Diptera: Sarcophagidae) is a large (15–16 mm body length) flesh fly common across England and Wales, but rarer in Scotland ( NBN Atlas Partnership, 2021), with a wider Palaearctic distribution ( Pape, 1996). Adults have been recorded from April to September, reaching peak abundance in July and August in the UK. As is typical among flesh flies, adult *S. variegata* are black to grey overall, with longitudinal stripes on the thorax and a checked pattern on the abdomen, and so reliable identification of this species using morphological characters alone is challenging. Separation of this species from the other members of the so-called *carnaria* subgroup ( *S. carnaria* and *S. subvicina*) is especially difficult, requiring examination of male terminalia and, for females, DNA barcoding ( Jordaens *et al.*, 2013; Schönberger *et al.*, 2022). Cuticular hydrocarbons have also recently been proposed as a possible identification method ( Moore *et al.*, 2021). *S. variegata* was described as *Musca variegata* by Giovanni Antonio Scopoli in 1763 in his *Entomologia Carniolica* ( Scopoli, 1763), and has in the past been regarded as a synonym of *S. carnaria* ( Van Emden, 1954).

Flesh flies are of forensic importantance ( Ren *et al.*, 2018), including their role as vectors for the transfer of contaminating DNA ( Durdle, 2020), and *S. variegata* adults have been reported to be attracted to beef liver baits and pig carcasses ( Anton *et al.*, 2011), and can be considered an indicators of summertime urban habitats ( Fremdt & Amendt, 2014). This *S. variegata* genome will be useful for the development of new molecular tools for species identification within this cryptic genus, for the investigation into the evolution of ovoviviparity; and as a resource for wider research into genome evolution in Diptera or insects more generally. Indeed, this genome sequence has already been used in an analysis of Hox cluster evolution in 243 insects ( Mulhair & Holland, 2022).

## Genome sequence report

**Figure 1.**
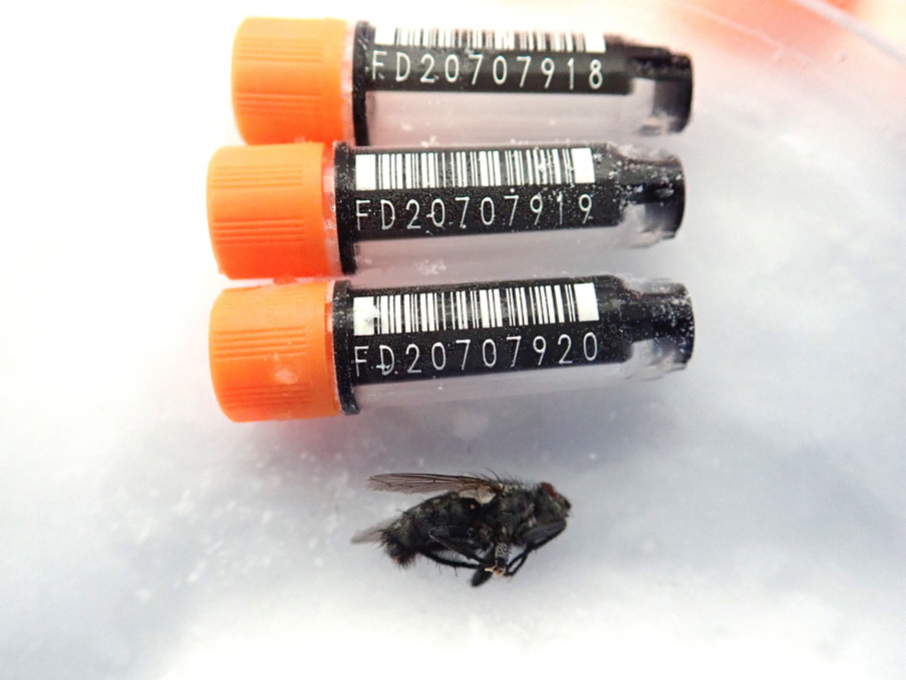
Photograph of the *Sarcophaga variegata* (idSarVari1) specimen used for genome sequencing.

The genome was sequenced from one male *Sarcophaga variegata* specimen ( Figure 1) collected from Wytham Woods, Oxfordshire, UK (latitude 51.77, longitude – 1.33). A total of 35-fold coverage in Pacific Biosciences single-molecule HiFi long reads and 71.0-fold coverage in 10X Genomics read clouds were generated. Primary assembly contigs were scaffolded with chromosome conformation Hi-C data. Manual assembly curation corrected 410 missing joins or mis-joins and removed one haplotypic duplication, reducing the scaffold number by 75.21%, and increasing the scaffold N50 by 157.09%.

**Figure 2.**
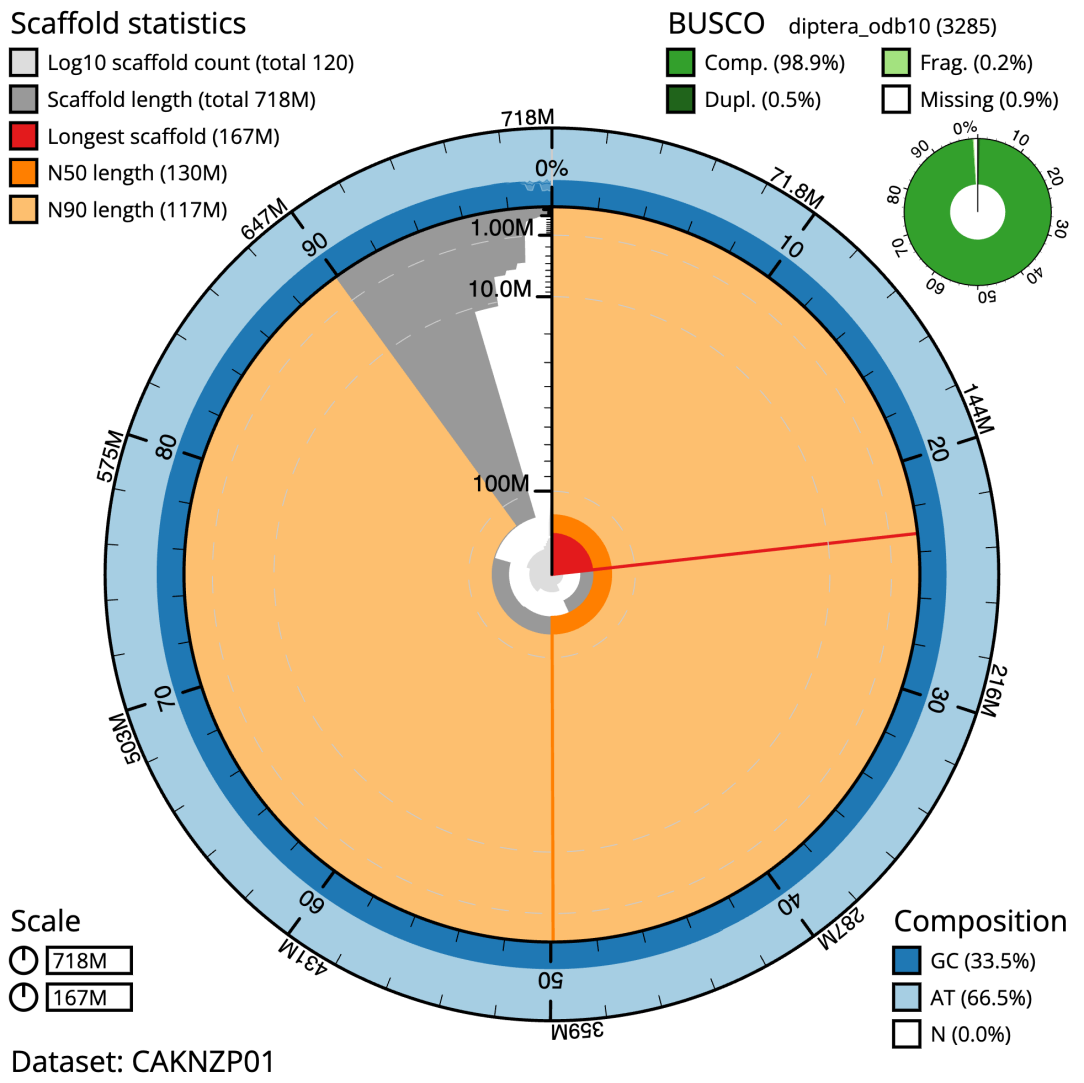
Genome assembly of *Sarcophaga variegata*, idSarVari1.1: metrics. The BlobToolKit Snailplot shows N50 metrics and BUSCO gene completeness. The main plot is divided into 1,000 size-ordered bins around the circumference with each bin representing 0.1% of the 718,474,625 bp assembly. The distribution of scaffold lengths is shown in dark grey with the plot radius scaled to the longest scaffold present in the assembly (167,104,922 bp, shown in red). Orange and pale-orange arcs show the N50 and N90 scaffold lengths (130,241,815 and 116,879,514 bp), respectively. The pale grey spiral shows the cumulative scaffold count on a log scale with white scale lines showing successive orders of magnitude. The blue and pale-blue area around the outside of the plot shows the distribution of GC, AT and N percentages in the same bins as the inner plot. A summary of complete, fragmented, duplicated and missing BUSCO genes in the diptera_odb10 set is shown in the top right. An interactive version of this figure is available at https://blobtoolkit.genomehubs.org/view/idSarVari1.1/dataset/CAKNZP01/snail.

The final assembly has a total length of 718.5 Mb in 119 sequence scaffolds with a scaffold N50 of 130.2 Mb ( Table 1). Most (98.85%) of the assembly sequence was assigned to 7 chromosomal-level scaffolds, representing 5 autosomes and the X and Y sex chromosomes. Chromosome-scale scaffolds confirmed by the Hi-C data are named in order of size ( Figure 2– Figure 5; Table 2). The order and orientation of scaffolds is uncertain in the following regions: chromosome 4: 53.2–62.5 Mb, and chromosome 5: 39.1–41.2 Mb. While not fully phased, the assembly deposited is of one haplotype. Contigs corresponding to the second haplotype have also been deposited. The mitochondrial genome was also assembled and can be found as a contig within the multifasta file of the genome submission.

**Table 1.** Genome data for *Sarcophaga variegata*, idSarVari1.1.

Project accession data		
Assembly identifier	idSarVari1.1	
Species	*Sarcophaga variegata*	
Specimen	idSarVari1	
NCBI taxonomy ID	236851	
BioProject	PRJEB48115	
BioSample ID	SAMEA8603132	
Isolate information	idSarVari1, male (genome sequencing and HiC)	
Assembly metrics *		*Benchmark*
Consensus quality (QV)	52.9	*≥ 50*
*k*-mer completeness	99.99%	*≥ 95%*
BUSCO **	C:98.9%[S:98.4%,D:0.5%], F:0.2%,M:0.9%,n:3,285	*C ≥ 95%*
Percentage of assembly mapped to chromosomes	98.85%	*≥ 95%*
Sex chromosomes	X and Y chromosomes	*localised homologous pairs*
Organelles	Mitochondrial genome assembled	*complete single alleles*
Raw data accessions		
PacificBiosciences SEQUEL II	ERR7123977, ERR7123978	
10X Genomics Illumina	ERR7113567–ERR7113570	
Hi-C Illumina	ERR7113566	
Genome assembly		
Assembly accession	GCA_932273835.1	
*Accession of alternate haplotype*	GCA_932276125.1	
Span (Mb)	718.5	
Number of contigs	602	
Contig N50 length (Mb)	7.4	
Number of scaffolds	119	
Scaffold N50 length (Mb)	130.2	
Longest scaffold (Mb)	167.1	
Genome annotation		
Number of protein-coding genes	16,660	
Number of non-coding genes	11,439	
Number of gene transcripts	38,740	

* Assembly metric benchmarks are adapted from column VGP-2020 of “Table 1: Proposed standards and metrics for defining genome assembly quality” from ( Rhie *et al.*, 2021). ** BUSCO scores based on the diptera_odb10 BUSCO set using v5.3.2. C = complete [S = single copy, D = duplicated], F = fragmented, M = missing, n = number of orthologues in comparison. A full set of BUSCO scores is available at https://blobtoolkit.genomehubs.org/view/idSarVari1.1/dataset/CAKNZP01/busco.

**Figure 3.**
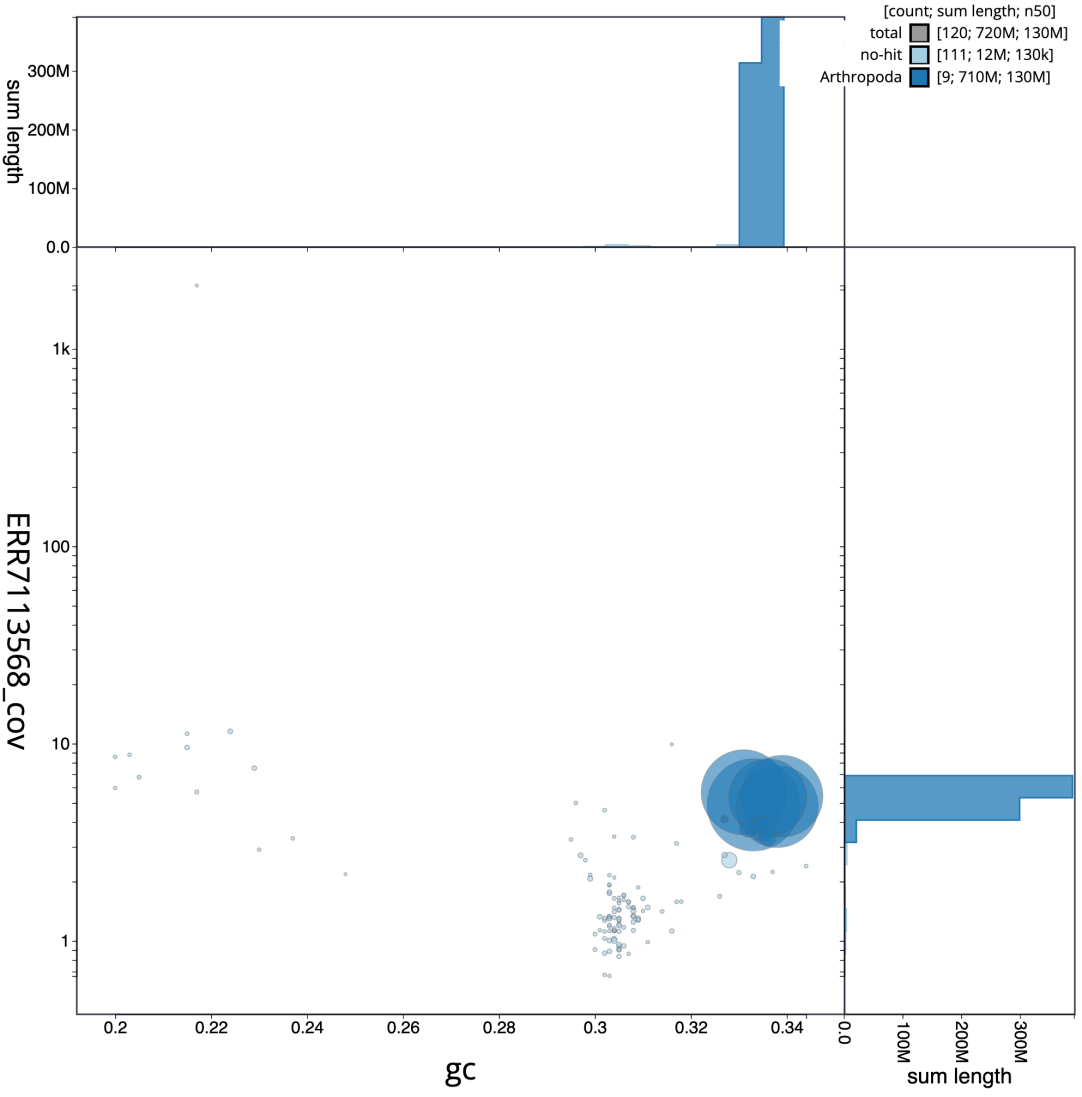
Genome assembly of *Sarcophaga variegata*, idSarVari1.1: BlobToolKit GC-coverage plot. Scaffolds are coloured by phylum. Circles are sized in proportion to scaffold length. Histograms show the distribution of scaffold length sum along each axis. An interactive version of this figure is available at https://blobtoolkit.genomehubs.org/view/idSarVari1.1/dataset/CAKNZP01/blob.

**Figure 4.**
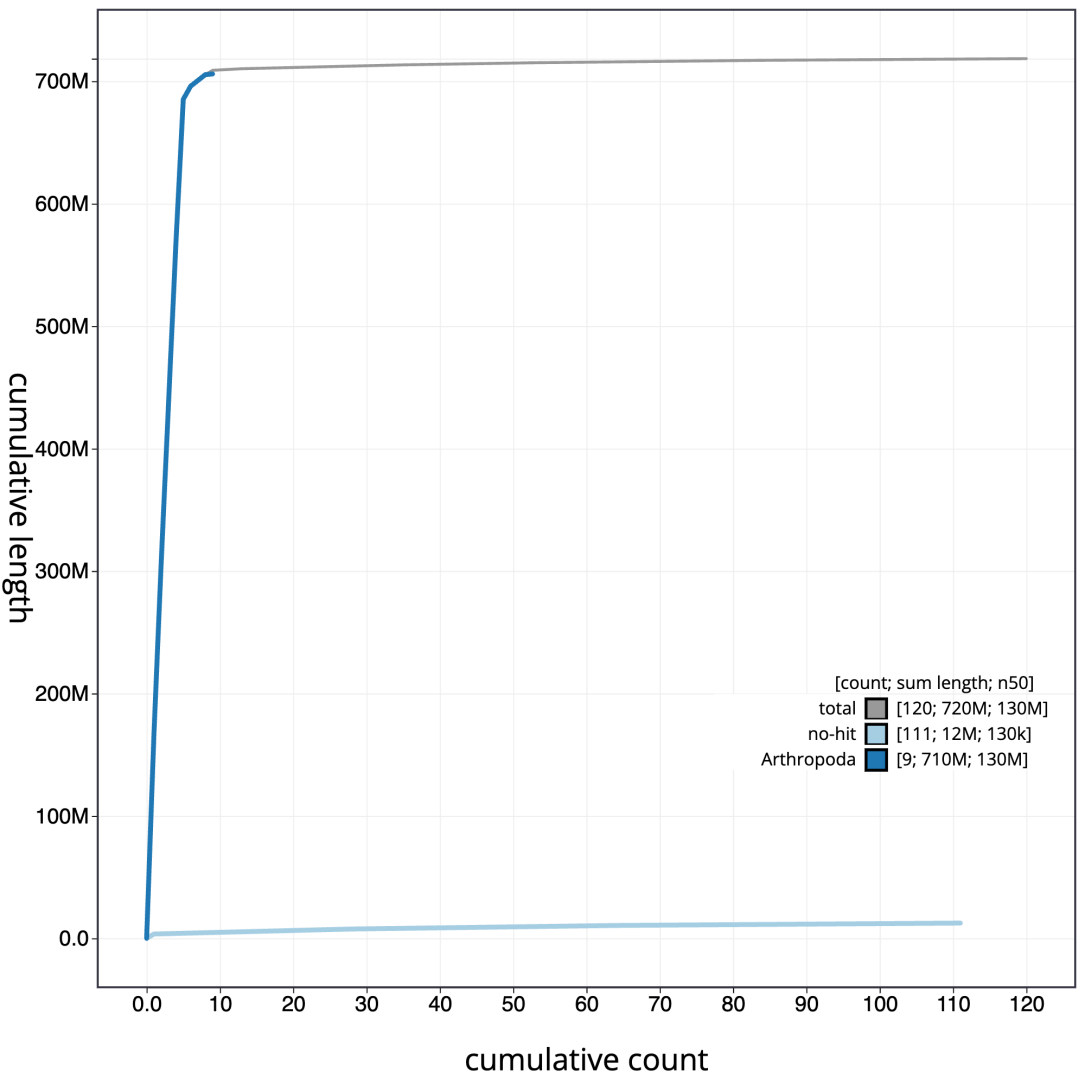
Genome assembly of *Sarcophaga variegata*, idSarVari1.1: BlobToolKit cumulative sequence plot. The grey line shows cumulative length for all scaffolds. Coloured lines show cumulative lengths of scaffolds assigned to each phylum using the buscogenes taxrule. An interactive version of this figure is available at https://blobtoolkit.genomehubs.org/view/idSarVari1.1/dataset/CAKNZP01/cumulative.

**Figure 5.**
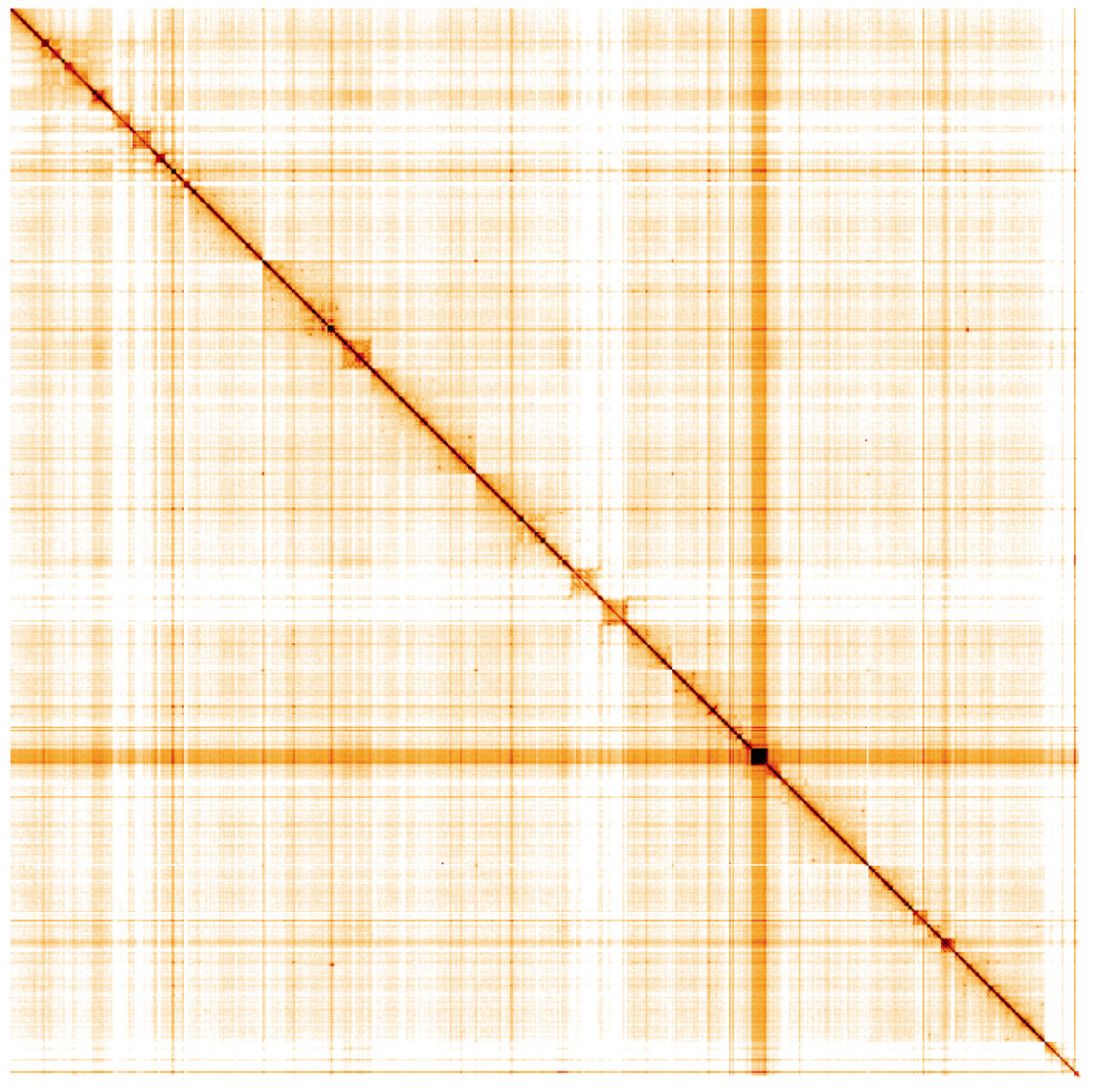
Genome assembly of *Sarcophaga variegata*, idSarVari1.1: Hi-C contact map of the idSarVari1.1 assembly, visualised using HiGlass. Chromosomes are shown in order of size from left to right and top to bottom. An interactive version of this figure may be viewed at https://genome-note-higlass.tol.sanger.ac.uk/l/?d=f8AEpuqjQR-5DIpdzaZLJA.

**Table 2.** Chromosomal pseudomolecules in the genome assembly of *Sarcophaga variegata*, idSarVari1.

INSDC accession	Chromosome	Size (Mb)	GC%
OW026358.1	1	167.11	33.3
OW026359.1	2	141.46	33.1
OW026360.1	3	130.24	33.8
OW026361.1	4	129.56	33.9
OW026362.1	5	116.88	33.6
OW026363.1	X	10.72	33.6
OW026364.1	Y	3.57	32.8
OW026365.1	MT	0.02	21.7
-	unplaced	18.92	31.7

The estimated Quality Value (QV) of the final assembly is 52.9 with *k*-mer completeness of 99.99%, and the assembly has a BUSCO v5.3.2 completeness of 98.9% (single = 98.4%, duplicated = 0.5%), using the diptera_odb10 reference set ( *n* = 3,285).

Metadata for specimens, spectral estimates, sequencing runs, contaminants and pre-curation assembly statistics can be found at https://links.tol.sanger.ac.uk/species/236851.

## Genome annotation report

The *S. variegata* genome assembly (GCA_932273835.1) was annotated using the Ensembl rapid annotation pipeline ( Table 1; https://rapid.ensembl.org/Sarcophaga_variegata_GCA_932276125.1/Info/Index). The resulting annotation includes 38,740 transcribed mRNAs from 16,660 protein-coding and 11,439 non-coding genes.

## Methods

### Sample acquisition and nucleic acid extraction

A male *Sarcophaga variegata* (idSarVari1) was collected from Wytham Woods, Oxfordshire (biological vice-county Berkshire), UK (latitude 51.77, longitude – 1.33) on 4 August 2020 by netting. The specimen was collected and identified by Steven Falk (independent researcher), and was then preserved on dry ice prior to processing. 

DNA was extracted at the Tree of Life laboratory, Wellcome Sanger Institute (WSI). The idSarVari1 sample was weighed and dissected on dry ice with tissue set aside for Hi-C sequencing. Thorax tissue was cryogenically disrupted to a fine powder using a Covaris cryoPREP Automated Dry Pulveriser, receiving multiple impacts. High molecular weight (HMW) DNA was extracted using the Qiagen MagAttract HMW DNA extraction kit. Low molecular weight DNA was removed from a 20 ng aliquot of extracted DNA using the 0.8X AMpure XP purification kit prior to 10X Chromium sequencing; a minimum of 50 ng DNA was submitted for 10X sequencing. HMW DNA was sheared into an average fragment size of 12–20 kb in a Megaruptor 3 system with speed setting 30. Sheared DNA was purified by solid-phase reversible immobilisation using AMPure PB beads with a 1.8X ratio of beads to sample to remove the shorter fragments and concentrate the DNA sample. The concentration of the sheared and purified DNA was assessed using a Nanodrop spectrophotometer and Qubit Fluorometer and Qubit dsDNA High Sensitivity Assay kit. Fragment size distribution was evaluated by running the sample on the FemtoPulse system.

### Sequencing

Pacific Biosciences HiFi circular consensus and 10X Genomics read cloud DNA sequencing libraries were constructed according to the manufacturers’ instructions. DNA sequencing was performed by the Scientific Operations core at the WSI on Pacific Biosciences SEQUEL II (HiFi) and Illumina NovaSeq 6000 (10X) instruments. Hi-C data were also generated from head tissue of idSarVari1 using the Arima2 kit and sequenced on the Illumina NovaSeq 6000 instrument.

### Genome assembly, curation and evaluation

Assembly was carried out with Hifiasm ( Cheng *et al.*, 2021) and haplotypic duplication was identified and removed with purge_dups ( Guan *et al.*, 2020). One round of polishing was performed by aligning 10X Genomics read data to the assembly with Long Ranger ALIGN, calling variants with FreeBayes ( Garrison & Marth, 2012). The assembly was then scaffolded with Hi-C data ( Rao *et al.*, 2014) using SALSA2 ( Ghurye *et al.*, 2019). The assembly was checked for contamination as described previously ( Howe *et al.*, 2021). Manual curation was performed using HiGlass ( Kerpedjiev *et al.*, 2018) and Pretext ( Harry, 2022). The mitochondrial genome was assembled using MitoHiFi ( Uliano-Silva *et al.*, 2022), which runs MitoFinder ( Allio *et al.*, 2020) or MITOS ( Bernt *et al.*, 2013) and uses these annotations to select the final mitochondrial contig and to ensure the general quality of the sequence.

A Hi-C map for the final assembly was produced using bwa-mem2 ( Vasimuddin *et al.*, 2019) in the Cooler file format ( Abdennur & Mirny, 2020). To assess the assembly metrics, the *k*-mer completeness and QV consensus quality values were calculated in Merqury ( Rhie *et al.*, 2020). This work was done using Nextflow ( Di Tommaso *et al.*, 2017) DSL2 pipelines “sanger-tol/readmapping” ( Surana *et al.*, 2023a) and “sanger-tol/genomenote” ( Surana *et al.*, 2023b). The genome was analysed within the BlobToolKit environment ( Challis *et al.*, 2020) and BUSCO scores ( Simão *et al.*, 2015; Manni *et al.*, 2021) were calculated.

Table 3 contains a list of relevant software tool versions and sources.

**Table 3.** Software tools: versions and sources.

Software tool	Version	Source
BlobToolKit	3.4.0	https://github.com/blobtoolkit/blobtoolkit
BUSCO	5.3.2	https://gitlab.com/ezlab/busco
FreeBayes	1.3.1-17-gaa2ace8	https://github.com/freebayes/freebayes
Hifiasm	0.15.3	https://github.com/chhylp123/hifiasm
HiGlass	1.11.6	https://github.com/higlass/higlass
Long Ranger ALIGN	2.2.2	https://support.10xgenomics.com/genome-exome/software/pipelines/latest/advanced/other-pipelines
Merqury	MerquryFK	https://github.com/thegenemyers/MERQURY.FK
MitoHiFi	2	https://github.com/marcelauliano/MitoHiFi
PretextView	0.2	https://github.com/wtsi-hpag/PretextView
purge_dups	1.2.3	https://github.com/dfguan/purge_dups
SALSA	2.2	https://github.com/salsa-rs/salsa
sanger-tol/genomenote	v1.0	https://github.com/sanger-tol/genomenote
sanger-tol/readmapping	1.1.0	https://github.com/sanger-tol/readmapping/tree/1.1.0

### Genome annotation

The Ensembl gene annotation system ( Aken *et al.*, 2016) was used to generate annotation for the *Sarcophaga variegata* assembly (GCA_932276125.1). Annotation was created primarily through alignment of transcriptomic data to the genome, with gap filling via protein-to-genome alignments of a select set of proteins from UniProt ( UniProt Consortium, 2019).

### Legal and ethical review process for Darwin Tree of Life Partner submitted materials

The materials that have contributed to this genome note have been supplied by a Darwin Tree of Life Partner. The submission of materials by a Darwin Tree of Life Partner is subject to the **‘Darwin Tree of Life Project Sampling Code of Practice’**, which can be found in full on the Darwin Tree of Life website here. By agreeing with and signing up to the Sampling Code of Practice, the Darwin Tree of Life Partner agrees they will meet the legal and ethical requirements and standards set out within this document in respect of all samples acquired for, and supplied to, the Darwin Tree of Life Project.

Further, the Wellcome Sanger Institute employs a process whereby due diligence is carried out proportionate to the nature of the materials themselves, and the circumstances under which they have been/are to be collected and provided for use. The purpose of this is to address and mitigate any potential legal and/or ethical implications of receipt and use of the materials as part of the research project, and to ensure that in doing so we align with best practice wherever possible.

The overarching areas of consideration are:

- Ethical review of provenance and sourcing of the material
- Legality of collection, transfer and use (national and international)

Each transfer of samples is further undertaken according to a Research Collaboration Agreement or Material Transfer Agreement entered into by the Darwin Tree of Life Partner, Genome Research Limited (operating as the Wellcome Sanger Institute), and in some circumstances other Darwin Tree of Life collaborators.

## Data Availability

European Nucleotide Archive: *Sarcophaga variegata*. Accession number PRJEB48115; https://identifiers.org/ena.embl/PRJEB48115. ( Wellcome Sanger Institute, 2022) The genome sequence is released openly for reuse. The *Sarcophaga variegata* genome sequencing initiative is part of the Darwin Tree of Life (DToL) project. All raw sequence data and the assembly have been deposited in INSDC databases. Raw data and assembly accession identifiers are reported in Table 1.

## Author information

Members of the University of Oxford and Wytham Woods Genome Acquisition Lab are listed here: https://doi.org/10.5281/zenodo.4789928.

Members of the Darwin Tree of Life Barcoding collective are listed here: https://doi.org/10.5281/zenodo.4893703.

Members of the Wellcome Sanger Institute Tree of Life programme are listed here: https://doi.org/10.5281/zenodo.4783585.

Members of Wellcome Sanger Institute Scientific Operations: DNA Pipelines collective are listed here: https://doi.org/10.5281/zenodo.4790455.

Members of the Tree of Life Core Informatics collective are listed here: https://doi.org/10.5281/zenodo.5013541.

Members of the Darwin Tree of Life Consortium are listed here: https://doi.org/10.5281/zenodo.4783558.

## References

[ref-1] Abdennur N Mirny LA : Cooler: Scalable storage for Hi-C data and other genomically labeled arrays. *Bioinformatics.* 2020;36(1):311–316. 10.1093/bioinformatics/btz540 31290943 PMC8205516

[ref-2] Aken BL Ayling S Barrell D : The Ensembl gene annotation system. *Database (Oxford).* 2016;2016:baw093. 10.1093/database/baw093 27337980 PMC4919035

[ref-3] Allio R Schomaker-Bastos A Romiguier J : MitoFinder: Efficient automated large‐scale extraction of mitogenomic data in target enrichment phylogenomics. *Mol Ecol Resour.* 2020;20(4):892–905. 10.1111/1755-0998.13160 32243090 PMC7497042

[ref-4] Anton E Niederegger S Beutel RG : Beetles and flies collected on pig carrion in an experimental setting in Thuringia and their forensic implications. *Med Vet Entomol.* 2011;25(4):353–364. 10.1111/j.1365-2915.2011.00975.x 21834830

[ref-5] Bernt M Donath A Jühling F : MITOS: Improved *de novo* metazoan mitochondrial genome annotation. *Mol Phylogenet Evol.* 2013;69(2):313–319. 10.1016/j.ympev.2012.08.023 22982435

[ref-6] Buenaventura E Pape T : Multilocus and multiregional phylogeny reconstruction of the genus *Sarcophaga* (Diptera, Sarcophagidae). *Mol Phylogenet Evol.* 2017;107:619–629. 10.1016/j.ympev.2016.12.028 28027962

[ref-7] Challis R Richards E Rajan J : BlobToolKit - interactive quality assessment of genome assemblies. *G3 (Bethesda).* 2020;10(4):1361–1374. 10.1534/g3.119.400908 32071071 PMC7144090

[ref-8] Cheng H Concepcion GT Feng X : Haplotype-resolved *de novo* assembly using phased assembly graphs with hifiasm. *Nat Methods.* 2021;18(2):170–175. 10.1038/s41592-020-01056-5 33526886 PMC7961889

[ref-33] Di Tommaso P Chatzou M Floden EW : Nextflow enables reproducible computational workflows. *Nat Biotechnol.* 2017;35(4):316–319. 10.1038/nbt.3820 28398311

[ref-9] Durdle A : Insects as vectors of DNA in a forensic context. *WIREs Forensic Science.* 2020;2(2). 10.1002/wfs2.1355

[ref-11] Fremdt H Amendt J : Species composition of forensically important blow flies (Diptera: Calliphoridae) and flesh flies (Diptera: Sarcophagidae) through space and time. *Forensic Sci Int.* 2014;236:1–9. 10.1016/j.forsciint.2013.12.010 24529768

[ref-12] Garrison E Marth G : Haplotype-based variant detection from short-read sequencing. 2012. 10.48550/arXiv.1207.3907

[ref-13] Ghurye J Rhie A Walenz BP : Integrating Hi-C links with assembly graphs for chromosome-scale assembly. *PLoS Comput Biol.* 2019;15(8):e1007273. 10.1371/journal.pcbi.1007273 31433799 PMC6719893

[ref-14] Guan D McCarthy SA Wood J : Identifying and removing haplotypic duplication in primary genome assemblies. *Bioinformatics.* 2020;36(9):2896–2898. 10.1093/bioinformatics/btaa025 31971576 PMC7203741

[ref-15] Harry E : PretextView (Paired REad TEXTure Viewer): A desktop application for viewing pretext contact maps. 2022; (Accessed: 19 October 2022). Reference Source

[ref-16] Howe K Chow W Collins J : Significantly improving the quality of genome assemblies through curation. *GigaScience.* Oxford University Press,2021;10(1):giaa153. 10.1093/gigascience/giaa153 33420778 PMC7794651

[ref-17] Jordaens K Sonet G Richet R : Identification of forensically important *Sarcophaga* species (Diptera: Sarcophagidae) using the mitochondrial *COI* gene. *Int J Legal Med.* 2013;127(2):491–504. 10.1007/s00414-012-0767-6 22960880

[ref-18] Kerpedjiev P Abdennur N Lekschas F : HiGlass: Web-based visual exploration and analysis of genome interaction maps. *Genome Biol.* 2018;19(1):125. 10.1186/s13059-018-1486-1 30143029 PMC6109259

[ref-19] Manni M Berkeley MR Seppey M : BUSCO Update: Novel and Streamlined Workflows along with Broader and Deeper Phylogenetic Coverage for Scoring of Eukaryotic, Prokaryotic, and Viral Genomes. *Mol Biol Evol.* 2021;38(10):4647–4654. 10.1093/molbev/msab199 34320186 PMC8476166

[ref-20] Moore HE Hall MJR Drijfhout FP : Cuticular hydrocarbons for identifying Sarcophagidae (Diptera). *Sci Rep.* 2021;11(1):7732. 10.1038/s41598-021-87221-y 33833323 PMC8032779

[ref-21] Mulhair PO Holland PWH : Evolution of the insect Hox gene cluster: Comparative analysis across 243 species. *Semin Cell Dev Biol.* 2022; S1084-9521(22)00357-3. 10.1016/j.semcdb.2022.11.010 36526530 PMC10914929

[ref-22] NBN Atlas Partnership: *Sarcophaga variegata* (Scopoli, 1763). *NBN Atlas.* 2021; (Accessed: 27 April 2023). Reference Source

[ref-23] Pape T : Catalogue of the Sarcophagidae of the world (Insecta: Diptera).Associated Publishers,1996. Reference Source

[ref-24] Rao SSP Huntley MH Durand NC : A 3D map of the human genome at kilobase resolution reveals principles of chromatin looping. *Cell.* 2014;159(7):1665–80. 10.1016/j.cell.2014.11.021 25497547 PMC5635824

[ref-25] Ren L Shang Y Chen W : A brief review of forensically important flesh flies (Diptera: Sarcophagidae). *Forensic Sci Res.* 2018;3(1):16–26. 10.1080/20961790.2018.1432099 30483648 PMC6197121

[ref-27] Rhie A McCarthy SA Fedrigo O : Towards complete and error-free genome assemblies of all vertebrate species. *Nature.* 2021;592(7856):737–746. 10.1038/s41586-021-03451-0 33911273 PMC8081667

[ref-26] Rhie A Walenz BP Koren S : Merqury: Reference-free quality, completeness, and phasing assessment for genome assemblies. *Genome Biol.* 2020;21(1):245. 10.1186/s13059-020-02134-9 32928274 PMC7488777

[ref-28] Schönberger D Giordani G Vanin S : A review of morphological characters for the identification of three common European species of *Sarcophaga* s. str.(Diptera: Sarcophagidae), with an emphasis on female terminalia. *Zootaxa.* 2022;5205(5):463–480. 10.11646/zootaxa.5205.5.4 37045422

[ref-29] Scopoli JA : Entomologia Carniolica exhibens insecta Carnioliae indigena et distributa in ordines, genera, species, varietates.In: *Methodo Linnaeana.*Trattner: Vindobonae,1763;1–421. Reference Source

[ref-30] Simão FA Waterhouse RM Ioannidis P : BUSCO: assessing genome assembly and annotation completeness with single-copy orthologs. *Bioinformatics.* 2015;31(19):3210–3212. 10.1093/bioinformatics/btv351 26059717

[ref-31] Surana P Muffato M Qi G : sanger-tol/readmapping: sanger-tol/readmapping v1.1.0 - Hebridean Black (1.1.0).Zenodo. 2023a; (Accessed: 17 April 2023). 10.5281/zenodo.7755665

[ref-32] Surana P Muffato M Sadasivan Baby C : sanger-tol/genomenote (v1.0.dev).Zenodo. 2023b; (Accessed: 17 April 2023). 10.5281/zenodo.6785935

[ref-34] Uliano-Silva M Ferreira JGRN Krasheninnikova K : MitoHiFi: a python pipeline for mitochondrial genome assembly from PacBio High Fidelity reads. *bioRxiv.* [Preprint],2022. 10.1101/2022.12.23.521667 PMC10354987 37464285

[ref-35] UniProt Consortium: UniProt: a worldwide hub of protein knowledge. *Nucleic Acids Res.* 2019;47(D1):D506–D515. 10.1093/nar/gky1049 30395287 PMC6323992

[ref-10] Van Emden FI : Diptera Cyclorrhapha, Calyptrata (I) Section (a). Tachinidae and Calliphoridae.In: *Handbooks for the identification of British insects.* in. Entomological Society of London,1954. Reference Source

[ref-36] Vasimuddin Md Misra S Li H : Efficient Architecture-Aware Acceleration of BWA-MEM for Multicore Systems.In: * 2019 IEEE International Parallel and Distributed Processing Symposium (IPDPS).*IEEE,2019;314–324. 10.48550/arXiv.1907.12931

[ref-38] Wellcome Sanger Institute: The genome sequence of the variegated flesh fly, *Sarcophaga variegata* (Scopoli, 1763). European Nucleotide Archive.[dataset], accession number PRJEB48115,2022.

[ref-37] Whitmore D Griffiths C Jones NP : New Sarcophagidae Recording Scheme. *Bulletin of the Dipterists Forum.* 2020;89:7–10. Reference Source

