# Supplementary material for: The genome sequence of the variegated flesh fly, Sarcophaga variegata (Scopoli, 1763)
Source: Wellcome Open Res. 2023 Jun 2;8:234. [Version 1] doi: 10.12688/wellcomeopenres.19483.1 (PMC10951566; doi:10.12688/wellcomeopenres.19483.1)
Supplement: Peer review history

# Reviewer report: Reviewer response for version 1

**DOI:** 10.21956/wellcomeopenres.21581.r74955

**Published:** 2024-03-19

**Authors:** Yuqiang Xi

**Affiliations:** 1 Department of Entomology, Henan Agricultural University, Zhengzhou, China

The overall writing of this paper is commendable. The genome sequence of Sarcophaga variegata (Scopoli, 1763) is anticipated to significantly enrich the field of fly phylogenomics. However, there are a couple of questions that require clarification:

 The assembled mitochondrial genome spans 18.7 kilobases, exceeding the typical size of insect mitogenomes (~15k). What factors contribute to the larger size of this mitogenome?

 Could you please provide a clear explanation of the Hox cluster?

Are sufficient details of methods and materials provided to allow replication by others?

Yes

Is the rationale for creating the dataset(s) clearly described?

Yes

Are the datasets clearly presented in a useable and accessible format?

Yes

Are the protocols appropriate and is the work technically sound?

Yes

Reviewer Expertise:

Insect phylogeny

I confirm that I have read this submission and believe that I have an appropriate level of expertise to confirm that it is of an acceptable scientific standard.

# Reviewer report: Reviewer response for version 1

**DOI:** 10.21956/wellcomeopenres.21581.r74951

**Published:** 2024-03-19

**Authors:** Alex Makunin

**Affiliations:** 1 Wellcome Sanger Institute, Hinxton, England, UK

The data note by Falk et al. describes the genome assembly for *Sarcophaga variegata, *which seems to be among the six chromosomal assemblies for *Sarcophaga *species available at the time of writing. The data is presented in a standardised way and the only issue I can see is that the sources of transcriptomic data for gene annotation were not indicated (I assume those were some public datasets).

 Similarly to the previous reviewer, I would appreciate if the chromosome names and grid were displayed in the Hi-C plot at Fig 5.

 It might be also useful to indicate that the blob plot at Fig 3 was generated from a subset of 10x data alignment to the reference genome. On the similar line, inclusion of mitochondrion in the blob plot makes it hard to see sex chromosomes below the autosomes - it seems there is at least one sex-linked scaffold that is similar in size to Y. 

 It is also worth noting that the reported chromosome number agrees with the typical 2n=12 karyotype for *Sarcophaga.*

Are sufficient details of methods and materials provided to allow replication by others?

Yes

Is the rationale for creating the dataset(s) clearly described?

Yes

Are the datasets clearly presented in a useable and accessible format?

Yes

Are the protocols appropriate and is the work technically sound?

Yes

Reviewer Expertise:

comparative genomics

I confirm that I have read this submission and believe that I have an appropriate level of expertise to confirm that it is of an acceptable scientific standard.

## References

[1] : The karyotype of the forensically important flesh flies (Diptera: Sarcophagidae), Mini Review.2022; 10.31219/osf.io/564ft 10.31219/osf.io/564ft

# Reviewer report: Reviewer response for version 1

**DOI:** 10.21956/wellcomeopenres.21581.r74945

**Published:** 2024-02-29

**Authors:** Daniel Doucet

**Affiliations:** 1 Natural Resources Canada, Sault Ste. Marie, Ontario, Canada

This report presents the first complete genome sequence of the flesh fly *Sarcophaga variegata*, a close relative of *S. carnaria*. As the Sarcophaga genus is extremely speciose, obtaining genomes from key species and eventually whole sub-genera will be useful the examine the drivers of speciation in this taxa.

 The report follows the standard structure established by the tree of life project, with the important tables and figures that describe the quality of the genome. As far as I can tell the data has been acquired using state of the art methods.

 One thing I noticed however is how unorganized the Hi-C contact map appears to be (Fig 5). I am more familiar with data generated in lepidoptera where cis interactions are dominant, resulting in clearly distinguishable chromosomal scaffolds on the contact map. Looking around other full genomes in the Wellcome Open website, I noticed that other dipteran genomes suffer from the same high frequency of trans interactions. I would like to authors to add a line or two of explanation about this phenomenon. I also think it would be helpful to add grey lines to delimit chromosomes on that figure (e.g. see ref [1])

Are sufficient details of methods and materials provided to allow replication by others?

Yes

Is the rationale for creating the dataset(s) clearly described?

Yes

Are the datasets clearly presented in a useable and accessible format?

Partly

Are the protocols appropriate and is the work technically sound?

Yes

Reviewer Expertise:

Insect cell biology and molecular biology

I confirm that I have read this submission and believe that I have an appropriate level of expertise to confirm that it is of an acceptable scientific standard, however I have significant reservations, as outlined above.

## References

[1] : The genome sequence of the tapered dronefly, Eristalis pertinax (Scopoli, 1763). *Wellcome Open Res* .2021;6: 10.12688/wellcomeopenres.17267.2 292 10.12688/wellcomeopenres.17267.2 36873714 PMC9975435
